# Autophagy Improves Inflammatory Response in Sepsis Accompanied by Changes in Gut Microbiota

**DOI:** 10.1155/2024/9550301

**Published:** 2024-10-18

**Authors:** La Wang, WenJia Wang, GuiTong Jiang, ZunLi Ke, RuiXi Luo, WeiYi Tian

**Affiliations:** ^1^Department of Immunology and Microbiology, School of Basic Medical Sciences, Guizhou University of Traditional Chinese Medicine, Guiyang, China; ^2^Cengong County People's Hospital, Kaili, China

**Keywords:** 16S rRNA sequencing, autophagy, inflammation, intestinal microbiota, sepsis

## Abstract

**Background:** Sepsis is defined as a life-threatening disease. Autophagy and the microbiome are increasingly connected with sepsis. The aim of this study was to investigate the protective effect of autophagy and the possible mechanisms.

**Methods:** The septic rat model was established by cecal ligation perforation (CLP). Rapamycin (Rap), 3-methyladenine (3-MA), and chloroquine (CQ) were administered to interfere autophagy. Western blot (WB) was used to detect the expression of key proteins in autophagy. Hematoxylin and eosin (H&E) staining and enzyme-linked immunosorbent assays (ELISAs) were used to identify the effect of autophagy on various organs. 16S ribosomal RNA gene sequencing was used to analyze the changes of the gut microbiota.

**Results:** Rap significantly upregulated the expression of key autophagy proteins, and 3-MA reduced the relative expression compared to the CLP group. The autophagic flux showed a corresponding trend. Interestingly, the autophagy inducer significantly decreased the mortality and the lipopolysaccharide (LPS) level in serum compared with the CLP group. Autophagy activation significantly improves the inflammatory response in sepsis. Histopathological sections showed that CLP destroyed the tight junctions between ileal epithelial cells, while autophagy induction reversed the damage. The sequencing results showed that autophagy activation increased the alpha diversity and alterted the composition and structure of gut microbiota. The abundance of Proteobacteria was markedly decreased in the Rap group, whereas Bacteroidetes was notably increased compared with the CLP group. Additionally, the protective effect of autophagy further changed the biomarkers in the microbial community. The top 35 functions in each sample were analyzed to obtain 18 genes including RNA synthesis, ATP binding and transport, chromosome assignment, osmotic polysaccharide transport, transcytosis, and methylation.

**Conclusion:** Autophagy is able to improve inflammation and may directly or indirectly regulate the microbiota of septic rats. Autophagy may be an important target for future clinical interventions in the treatment of sepsis.

## 1. Introduction

Sepsis, a life-threatening organ dysfunction caused by a dysregulated host response to infection, is a leading cause of death in intensive care units [1]. Sepsis is caused by multiple bacterial infections and may progress to septic shock, resulting in extremely high mortality rates [2]. Sepsis has caused over 5 million deaths worldwide, making it a global health priority announced by the World Health Assembly and the World Health Organization in 2017 [3]. Although sepsis is very common and the mortality rate is very high, there are still no specific therapies for sepsis except targeted antimicrobial therapy [4].

Autophagy acts as an evolutionarily highly conserved degradation system induced by various triggers to maintain intracellular homeostasis [5]. The formation of autophagosomes, autophagosome–lysosome fusion, and the degradation of products are some of the steps in autophagy. As an immune mechanism, autophagy plays an important role in various inflammatory and infection-related diseases [6]. Autophagy has long been recognized as a cellular protective mechanism that limits cell damage and apoptosis in sepsis [7]. On the one hand, the activation and induction of autophagy protects the host against multiple organ dysfunction syndrome (MODS) by maintaining the number of immune cells and the balance of inflammatory cytokines [8]. On the other hand, impaired autophagy aggravates tissue and organ injury in sepsis [9]. Multiple strategies have been used to restore immune system homeostasis by modulating autophagy during sepsis [10]. Thus, exploring the role of autophagy in sepsis may provide some new ideas for its treatment in the future [11]. Autophagy, as many studies have shown, can not only remove proteins and organelles to be degraded but also eliminates pathogenic bacteria [12]. Thus, whether changes in autophagy alter the composition of the intestinal microbiota is an attractive topic.

The intestinal microbiota affects the host's susceptibility and response to sepsis through various pathways, and once destroyed, it can cause organ function damage [13]. Conversely, sepsis caused by multiple bacterial infections can also induce inflammation and oxidative stress pathways in the intestine, thereby reducing the proportion of beneficial bacteria in the intestine and damaging intestinal epithelial integrity [14]. In septic mice, the abundance of *Bacteroidota* was increased, while that of Firmicutes (Bacillota) was decreased at the phylum level. At the genus level, the abundance of Alistipes was increased, while that of Lachnospiraceae was decreased in mice with sepsis. The Firmicutes (Bacillota)/*Bacteroidota* (F/B) ratio was decreased in mice with sepsis when compared to that of control mice [15]. Research has shown that the impairment in autophagy in intestinal epithelial cells can also alter the diversity of gut microbiota, leading to immune dysfunction [16]. A similar study has shown that ATG16L deficiency accelerates the progression of multiple bacterial infections in sepsis [17]. Therefore, we speculated that there is a correlation between autophagy and intestinal microbiota homeostasis. Further studies are needed to design better therapeutic molecules to target sepsis via autophagy and the microbiota [11].

To investigate the effect of autophagy on the intestinal microbiota in septic rats, we used an autophagy inducer and inhibitors after cecal ligation perforation (CLP) to determine the entire process of autophagy from the formation of autophagosomes to the fusion of autophagosomes–lysosomes in sepsis. Then, the death of rats was recorded, and the blood, various tissues, and feces were collected at the appropriate time. The survival rate, cytokine levels, histopathology, and autophagy-related proteins (ATGs) were detected to demonstrate that autophagy was crucial in inflammatory regulation and protection against sepsis. To determine whether changes in the autophagy level in sepsis would affect the intestinal microbiota, we performed 16S ribosomal RNA (rRNA) gene sequencing analysis of feces. The results showed that autophagy intervention could significantly affect the diversity and physiological function of the gut microbiota in septic animals. In conclusion, this study demonstrates that autophagy has a significant regulatory effect on the homeostasis of the intestinal microbiota and thus has a protective effect on sepsis.

## 2. Materials and Methods

### 2.1. Animals and Ethical Approval

Male Sprague‒Dawley (SD) rats weighing 150–180 g were purchased from Hubei Experimental Animal Research Center in Hubei, China (Laboratory Animal Quality certificate no. 42000600042413). All rats were acclimated for 1 week before the experiments. All animals had free access to chow and water throughout the experimental period. All rats were kept at room temperature (20–26°C) with ~40%–60% humidity on a 12 h light−12 h dark cycle. No deaths occurred before the intervention. All experimental protocols were approved by the Hubei Experimental Animal Research Center (Wuhan, Hubei, China; ethical approval number: 2020-0018).

### 2.2. Sepsis Model

The CLP model was used to model sepsis as in previously published studies [18]. Briefly, rats were anesthetized with 3% pentobarbital (1 mL/mg), and a 2-cm midline incision was made. The cecum was fully exposed through the incision and ligated below the ileocecal valve. Then, the cecum was penetrated with a 20-gauge needle to obtain two pinholes, and a small amount of feces was extruded out of the pinhole. Finally, we placed the intestine back within the abdominal cavity and sutured the skin wounds layer by layer. The animals in the sham group were anesthetized and operated without ligation and puncture. Postoperatively, 3 mL of prewarmed sterile saline was administered subcutaneously (pyrogen-free 0.9% NaCl, 37°C) for fluid resuscitation. Animals were monitored continuously following anesthesia and then every 15 min until they were able to walk. A heating pad was placed under the recovery cage to prevent hypothermia.

### 2.3. Animal Grouping and Intervention Measures

Two independent experiments were conducted in our study. In the first experiment, rats were anesthetized at 24 h post-CLP to collect blood and various tissues for various detection (*n* = 5 for each group), including histopathological examination (HE), enzyme-linked immunosorbent assay (ELISA), and western blotting (WB). In the second experiment, seven rats were used in each group for mortality statistics and fecal collection. All rats were randomly divided into five groups: sham, CLP, CLP + rapamycin (Rap), CLP + 3-Methyladenine (3-MA), and CLP + chloroquine (CQ). The four CLP groups (except the sham group) were subjected to cecal ligation and puncture as described above. According to previous studies, Rap activated autophagy by binding with FKBP-12 to inhibit mTOR (mammalian target of rapamycin) C1 signaling [19]. Rap was dissolved in DMSO to 10 mM and then prepared with physiological saline to 3 mg/kg for intraperitoneal injection into animals. 3-MA is an inhibitor of autophagy via its inhibitory effect on class III PI3K. 3-MA was dissolved in DMSO to 50 mM and then prepared with physiological saline to 30 mg/kg for intraperitoneal injection [20]. CQ is an autophagy and toll-like receptor (TLR) inhibitor that was dissolved in DMSO to 100 mM and then diluted with physiological saline to prepare 50 mg/kg for intraperitoneal injection into animals at 1 h after CLP operation [21]. All drugs were purchased from MedChemExpress (MCE) and used according to the instructions.

### 2.4. Histopathological Examination

Multiple tissues including the heart, liver, lungs, spleen, kidney, and ileum were collected at 24 hr post-CLP and fixed in 4% paraformaldehyde. Paraffin sections (4 μm) were stained with hematoxylin and eosin (H&E) according to standard methods as previously reported [22]. The tissue sections were observed and photographed using a microscope (Leica microscope DM4B) and photomicrography image processing software (Nikon H500S) at magnifications of ×100 or ×200.

### 2.5. ELISA

The experimental rats were anesthetized via intraperitoneal injection with 3% pentobarbital (1 mL/mg) at 24 h. Blood was collected through the abdominal aorta. An endotoxin ELISA kit (Uscn Life Science, Wuhan, China) was used to measure the level of lipopolysaccharide (LPS) according to the instructions provided by the manufacturer. Other ELISA kits (Elabscience Biotechnology Co., Ltd, Wuhan, China) were used for the detection of tumor necrosis factor-*α* (TNF-*α*; E-EL-R2856c), interleukin-1*β* (IL-1*β*; E-EL-R0012C), interleukin-6 (IL-6; E-EL-R0015c), and interleukin-10 (IL-10; E-EL-R0016c) levels to understand the changes in cytokines in the serum of rats in each group.

### 2.6. WB

Tissues were collected at 24 h post-CLP for WB analysis of the proteins Microtubule-associated protein light chain 3 (MAP1LC3), Beclin 1, and p62. Total proteins were extracted using RIPA lysis and extraction buffer (Beyotime, China) containing protease inhibitor (Beyotime, China). Equivalent amounts of protein were separated via sodium dodecyl sulfate polyacrylamide gel electrophoresis (SDS-PAGE) (12% gel) and transferred onto polyvinylidene difluoride (PVDF) membranes. After blocking with 5% nonfat milk solution for 1 h at room temperature, the membranes were then incubated with primary antibody at 4°C overnight followed by washing with TBS(Tris buffered saline) with Tween-20 (TBST) (0.1%, v/v) five times for 10 min per wash. Finally, the membranes were incubated with horseradish peroxidase-conjugated secondary antibody for 1 h at room temperature followed by washing with TBST. The dilution ratio of the primary antibody was as follows: MAP1LC3 (Affbiotech, AF5402), 1:1000; Beclin 1 (Affbiotech, AF5128), 1:1000; p62 (Affbiotech, AF5384), 1:1000; and glyceraldehyde 3 phosphate dehydrogenase (GAPDH; Hangzhou Xianzhi Biology Co., Ltd, AB-P-R 001), 1:1000. GAPDH served as a loading control, and protein bands were quantified using ImageJ Software. The antibody–antigen complexes were visualized via chemiluminescence with an enhanced enhanced chemiluminescence (ECL) immunoblotting system (Beijing Pulilai Gene Technology Co., Ltd, P1050).

## 3. 16S rRNA Gene Sequencing and Bioinformatic Analysis

Fecal samples were collected 24 h post-CLP before euthanasia and frozen at −80°C. The total deoxyribonucleic acid (DNA) of the feces was extracted using the QIAamp PowerFecal DNA Kit (Qiagen, USA) according to the instructions provided by the manufacturer. The integrity of DNA was detected by agarose gel electrophoresis, and the concentration of DNA in each sample was detected by QubitTM3 Fluorometer (Invitrogen, USA). The 16S rRNA V4 region was amplified using specific primers: 515F (5′-GTGCCAGCMGCCGCGGTAA-3′) and 806R (5′-GGACTACHVGGGTW TCTAAT-3′) [23]. All polymerase chain reactions (PCRs) were carried out with Phusion High-Fidelity PCR Master Mix (New England Biolabs). Subsequently, PCR products were mixed in equidensity ratios. Then, mixed PCR products were purified with a Qiagen Gel Extraction Kit (Qiagen, USA). The library quality was assessed on a Qubit 2.0 Fluorometer (Thermo Scientific, USA). The libraries were sent for sequencing using the NovaSeq PE250 (Illumina, USA), which was conducted by Novogene Bioinformatics Technology Co., Ltd. (Beijing, China) [24].

The raw data are first concatenated and filtered to obtain effective data (clean data); then, DADA2 was used for noise reduction, which no longer clustered by similarity but only performed dereplication or equivalent to clustering with 100% similarity to obtain the final amplicon sequence variants (ASVs). For the obtained ASVs, on the one hand, species annotation is performed on the representative sequences of each ASV. At the same time, abundance, alpha diversity calculations, Venn plots, and petal plots were performed on ASVs to obtain information on species richness and evenness within the sample, as well as common and unique ASVs information between different samples or groups. Recombination detection program (RDP) was used to classify the sequences and to conduct analyses of alpha and beta diversity [25]. On the other hand, multiple sequence alignment was performed on ASVs to construct a phylogenetic tree. Differences in community structure between different samples or groups were explored through dimensionality reduction analysis such as principal coordinate analysis (PCoA) and sample clustering tree display. To further explore the differences in community structure among grouped samples, statistical analysis methods such as *t* test, metastat, and LDA effect size (LEfSe) were used to conduct significance tests on the species composition and community structure of grouped samples. PICRUSt2 software was used for functional prediction analysis of microbial communities in ecological samples.

### 3.1. Statistical Analysis

The data obtained in the research are presented as the mean ± SE, except for the survival rate. SPSS 18.0 software was used for statistical analysis. The statistical significance of differences was assessed with Student's *t* test for two groups or one-way analysis of variance (ANOVA) for multiple groups. The nonparametric Kruskal–Wallis test was used in the diversity analysis of intestinal microbiota. These analyses were performed using Prism V.6.0 (GraphPad, San Diego, CA, USA). *p* < 0.05 and *p* < 0.01 denoted statistical significance and extremely significant differences, respectively.

## 4. Results

### 4.1. Effect of Drug Treatment on Autophagy Levels in Multiple Organs in Septic Rats

**Figure 1:**
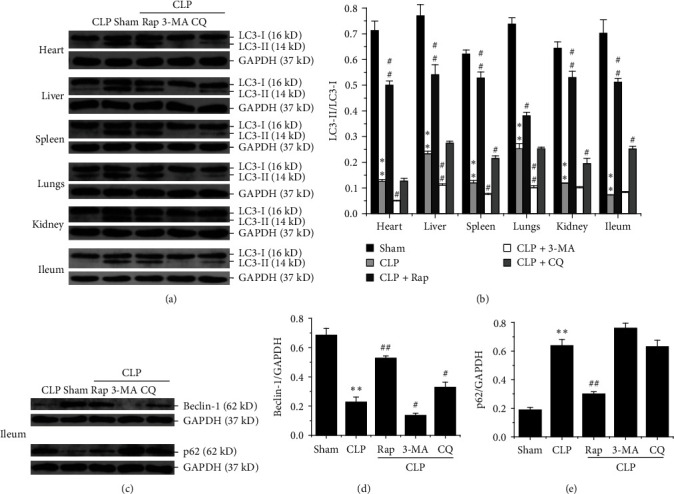
Detection of the effectiveness of autophagy regulators in septic rats. The rats were divided into five groups (sham control, CLP, CLP + Rap, CLP + 3-MA, and CLP + CQ). 3-MA (30 mg/kg MCE), Rap (3 mg/kg MCE), and CQ (50 mg/kg MCE) were administered by intraperitoneal injection 1 h after the CLP operation. (A) The expression of LC3-II in various organs of rats was detected by WB at 24 h post-CLP operation. (B) Representative results are shown with graphs representing the intensity of LC3-II/GAPDH. (C) The expression of Beclin 1 and p62 in the ileum was detected by WB at 24 h after CLP operation. (D, E) Representative results are shown with graphs representing the intensity of Beclin 1/GAPDH (D), and p62/GAPDH (E). The values are expressed as the means ± SEMs (*n* = 7 per group). Statistical significance was analyzed with Student's *t* test (*⁣*^*∗*^*p* < 0.05 and *⁣*^*∗∗*^*p* < 0.01, *⁣*^*∗*^, versus the sham group; ^#^*p* < 0.05 and ^##^*p* < 0.01, ^#^, versus the CLP group). 3-MA, 3-methyladenine; CLP, cecal ligation and puncture; CQ, chloroquine; GAPDH, glyceraldehyde 3 phosphate dehydrogenase; MCE, MedChemExpress; Rap, rapamycin; WB, western blotting.

Autophagy intervention drugs were administered intraperitoneally at appropriate concentrations at 1-h post CLP operation. Then, autophagy levels were detected to confirm drug efficacy. The lipidated form of MAP1LC3 (LC3-II) is an autophagosome-specific membrane marker, and it is widely used to determine the level of autophagy. The CLP model group had significantly lower LC3-II levels than the sham operation group in multiple organs, suggesting that autophagy had a certain protective effect in sepsis (Figure 1A,C). The CLP + Rap group showed significantly higher expression levels of LC3-II than the CLP group at 24 h postintervention (Figure 1A). In contrast, the autophagy suppression groups had obviously lower expression levels of LC3-II (Figure 1A). Statistical results also showed the same trend (Figure 1B). Moreover, the above changes generally existed in various organs of rats, suggesting the importance of autophagy and the effectiveness of drug treatment.

Subsequently, we selected ileal tissues for the further detection of other ATGs. Beclin 1, also known as ATG6, mediates autophagosome nucleation. The p62 is the first discovered autophagy selective receptor and an indicator for assessing autophagic flux [26]. Therefore, we detected Beclin 1 and p62 proteins by immunoblotting. The results revealed a significant increase in Beclin 1 and reduction in p62 in the CLP + Rap group compared with the CLP group (Figure 1C), which suggested that the level of autophagy flow increased, accompanied by good fusion and degradation of autophagosomes and lysosomes. Moreover, the CLP + 3-MA and CLP + CQ groups showed the opposite results compared with the CLP group (Figure 1C). The ratios of Beclin 1 or p62 to GAPDH indicated the same trend (Figures 1D,E).

### 4.2. Intervention in Autophagy Influenced Mortality, Inflammation, and Multiple Organ Injuries in Septic Rats

**Figure 2:**
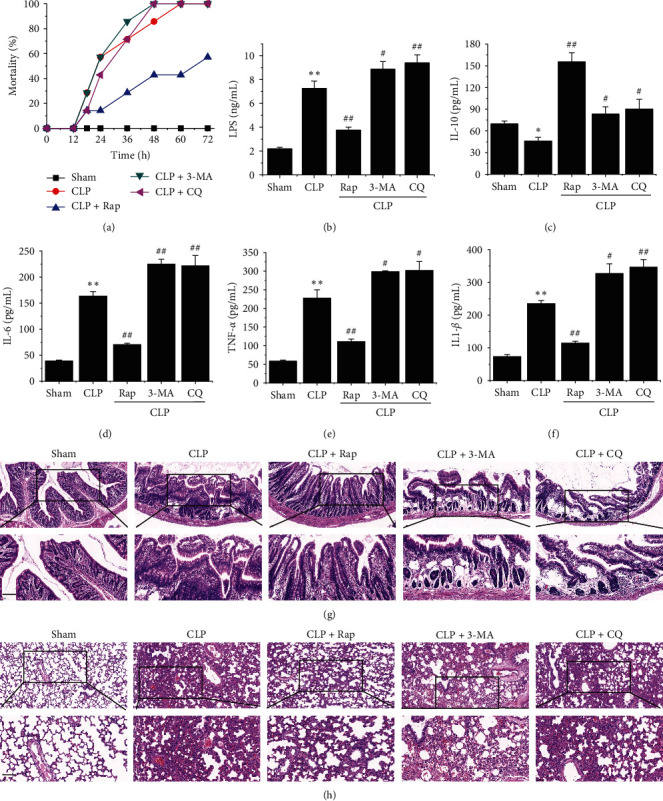
Intervention in autophagy influenced inflammation and multiple organ injuries in septic rats. (A) Change in the mortality of each group. (B) Detection of serum LPS in each group. (C–F) Detection of the levels of cytokines (IL-10, IL-6, IL-1*β*, and TNF-*α*) in the sera of each group using ELISA. (G, H) Histological appearance of the ileum and lung in the five groups after H&E staining (upper panel × 100 and lower panel × 200). The values are expressed as the means ± SEMs (*n* = 7 per group). Statistical significance was analyzed with Student's *t* test (*⁣*^*∗*^*p* < 0.05 and *⁣*^*∗∗*^*p* < 0.01, *⁣*^*∗*^, versus the sham group; ^#^*p* < 0.05 and ^##^*p* < 0.01, ^#^, versus the CLP group). ELISA, enzyme-linked immunosorbent assay; H&E, hematoxylin and eosin; IL-1*β*, interleukin-1*β;* IL-6, interleukin-6; IL-10, interleukin-10; LPS, lipopolysaccharide; TNF-*α*, tumor.

After CLP, the rats showed increased breathing and reduced consumption of food and water, dull fur, bloody discharge at the corner of the eye, and loose stool. Compared with the model group (100%), the autophagy induction group had a significantly lower mortality rate (60%), while autophagy inhibition accelerated the death of septic rats (Figure 2A). LPS, a component of the outer wall of gram-negative bacteria, can reflect the degree of bacterial infection to a certain extent. We detected the content of LPS in the serum of animals in each group by ELISA. The results showed that the autophagy induction group had a significantly reduced content of LPS in the serum of model animals, while autophagy inhibition exacerbated the infection of gram-negative bacteria to some extent (Figure 2B). The detection of inflammation-related cytokines showed that autophagy induction effectively increased the expression of the anti-inflammatory factor IL-10 (Figure 2C) and significantly reduced the levels of proinflammatory factors (IL-6, TNF-*α*, and IL-1*β;*Figure 2D–F). The results of H&E staining showed severe intestinal epithelial cell detachment, tissue congestion, and inflammatory exudation in the CLP group (Figure 2G). A certain degree of recovery in the autophagy induction group was observed, but it was more serious in the autophagy inhibition group (Figure 2G). Similarly, the lungs of septic rats showed severe fibrosis and tissue damage, increased alveolar wall thickness, pulmonary interstitial edema, a large amount of inflammatory cell infiltration, and other pathological changes (Figure 2H). Autophagy induction can effectively improve the injury state, while autophagy inhibition aggravates its pathological degree to a certain extent (Figure 2H). Interestingly, autophagy can improve pathological damage in septic rats, which is a systemic remission phenomenon, including in the heart, kidney, spleen, and liver (Supporting Information 1: Figure S1).

### 4.3. The Protection of Autophagy on Sepsis Altered Gut Microbial Richness and Diversity

To study the change of the gut microbiota in sepsis, we collected feces to analyze the microbiome by deep sequencing. An alpha rarefaction curve and species accumulation boxplot were constructed, and the flatter curve indicated sufficient reads collected from each sample (Supporting Information 1: Figure S2 A–C). The coverage rate of sequencing of the five groups was 100%, which indicated that all of the diversity was captured in all samples (Supporting Information 1: Figure S2 G). The richness of the microbial community expressed by the Chao1 index showed that CLP significantly lowered the community richness, while the CLP + Rap group reversed the trend to some extent compared with the other CLP groups (Supporting Information 1: Figure S2 D and Table 1). The alpha diversity was evaluated by the Simpson and Shannon indices. The two indices were significantly decreased in the CLP group compared with the sham group (Supporting Information 1: Figure S2 E–F and Table 1). The Shannon index showed that microbial diversity was significantly increased in the CLP + Rap group (Supporting Information 1: Figure S2 E and Table 1). Although no significant difference was observed, the Simpson index of the CLP + Rap group still showed an upward trend compared with that of the CLP group (Supporting Information 1: Figure S2 F and Table 1). Furthermore, the inhibition of autophagy or autophagic flux affected the microbial community richness and the alpha diversity of the intestinal microbiota in septic rats (Supporting Information 1: Figure S2 and Table 1). All of the above results indicated that the diversity of the intestinal microbiota in septic rats decreased significantly, and intervention of autophagy could substantially protect the balance of the intestinal microbiota.

### 4.4. The Protection of Autophagy on Sepsis Influenced the Composition and Structure of the Microbial Community

**Figure 3:**
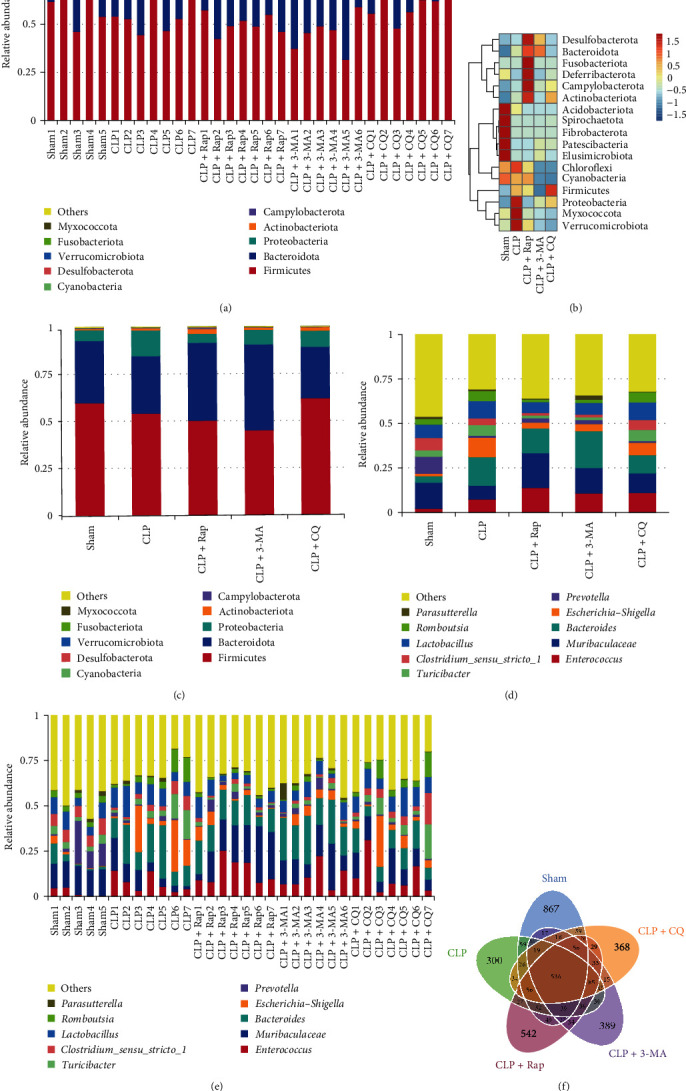
The protection of autophagy on sepsis influenced the richness and abundance of the microbial community. The top 10 species with the highest abundances in each sample (A) or group (C) at the phylum level. (B) Grouping clustering of bacteria at the phylum level. The top 10 taxa with the highest abundances in each sample (E) or group (D) at the genus level. (F) Venn diagram of shared and unique ASVs among the different groups. ASVs, amplicon sequence variants.

The community richness analysis and abundance clustering at the phylum level showed that there were 17 phyla of rat intestinal bacteria, and the abundances of Firmicutes, *Bacteroidota*, and Proteobacteria ranked in the top three in all five groups (Figure 3A–C). The grouping clustering results showed that the abundance of Proteobacteria was markedly increased, whereas Firmicutes and Bacteroidetes were notably decreased in the CLP group compared with the sham group (Figure 3B,C). At the genus level, the gut microbiota in the CLP group was mainly composed of *Bacteroides*, *Escherichia–Shigella*, *Lactobacillus*, *Enterococcus*, Muribaculaceae, *Romboutsia*, *Turicibacter*, and *Clostridium*_*sensu_stricto_1*, whlie Muribaculaceae, *Bacteroides*, *Enterococcus*, *Lactobacillus*, and *Escherichia–Shigella* in the CLP + Rap group. The abundance of *Bacteroides* and *Escherichia–Shigella* was increased, while that of Muribaculaceae and Prevotella was decreased in septic rats (Figure 3D,E). Autophagy activation significantly changed the abundance of microbiota at the phylum and genus levels caused by CLP, while autophagy inhibitors, especially the inhibition of autophagic flux, had no such effect (Figure 3B–E). According to the results of annotation, we selected the top 10 taxa with the highest abundances in each sample or group at each classification level (phylum, class, order, family, genus, and species) (Supporting Information 1: Figure S3 and Figure 3A–E). The result was consistent with the above. A flower diagram was drawn to describe the comparison and distribution of ASVs. The diagram showed that there were 867, 300, 542, 389, and 368 unique ASVs in the sham, CLP, CLP + Rap, CLP + 3-MA, and CLP + CQ group, respectively (Figure 3F). The flower diagram further confirmed the conclusion that the reduction in diversity was caused by CLP and that autophagy protection increased the diversity of the intestinal microbiota (Figure 3F). Thus, these results revealed that the host autophagy level might play a vital role in shaping the gut microbiome of septic rats.

**Figure 4:**
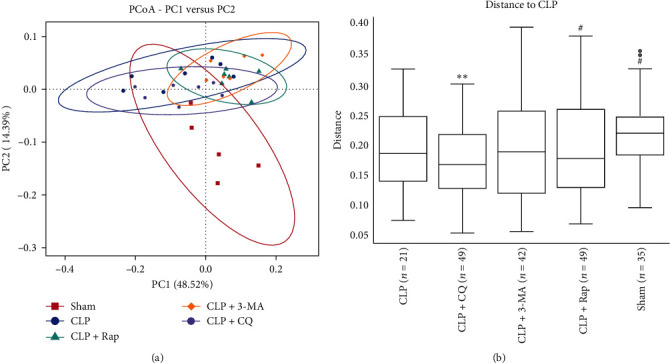
The protection of autophagy on sepsis influenced the microbial community composition. (A) PCoA of the beta diversity in the microbial communities in each group. (B) ANOSIM analysis of the unweighted UniFrac distance in each group. Statistical significance was analyzed with Student's *t* test (*⁣*^*∗*^*p* < 0.05 and *⁣*^*∗∗*^*p* < 0.01, *⁣*^*∗*^, versus the sham group; ^#^*p* < 0.05 and ^##^*p* < 0.01, ^#^, versus the CLP group). PCoA, principal coordinate analysis.

To understand the effect of autophagy protection on the intestinal microbiota profile of septic rats, PCoA was performed based on the ASVs to analyze beta diversity. It can be seen that the three CLP groups and the sham group were obviously separated (Figure 4A). This result indicated that the composition of the intestinal microbiota of the septic rats changed significantly after the model was established. Autophagy protection can partially regulate changes in the intestinal microbiota profile, but this adjustment is not very significant. Analysis of similarities (ANOSIM) of unweighted UniFrac distance showed that there were significant differences in the community structure between the sham group and the CLP group and between the CLP group and the CLP + Rap group (Figure 4B). Interestingly, there were also significant differences in the community structure between the CLP + 3-MA group and the CLP group, which may indicate that the changes in autophagy, whether activated or inhibited, were equally important to the intestinal microbiota of septic rats (Figure 4B). These results further demonstrated that the intestinal microbiota composition of the CLP groups changed significantly, while the autophagy protection could inhibit the abnormal microbiota to some extent.

### 4.5. The Protection of Autophagy on Sepsis Altered the Biomarkers in the Microbial Community

To find biomarkers with significant differences between groups, we used *t* tests and metastat to analyze the significance of the obtained data at the genus level (Supporting Information 1: Figure S4, Supporting Information 2: Table S1, and Supporting Information 3: Table S2). Through pairwise comparison between groups using *t* tests, we found that there were 32, 13, 7, and 18 genus with significant differences in the sham and CLP groups, the CLP and CLP + Rap groups, the CLP and CLP + 3-MA groups, and the CLP and CLP + CQ groups, respectively (Supporting Information 1: Figure S4 and Supporting Information 2: Table S1). Metastat analysis showed 94, 35, 27, and 56 genus with significant differences, respectively (Supporting Information 2: Table S2).

**Figure 5:**
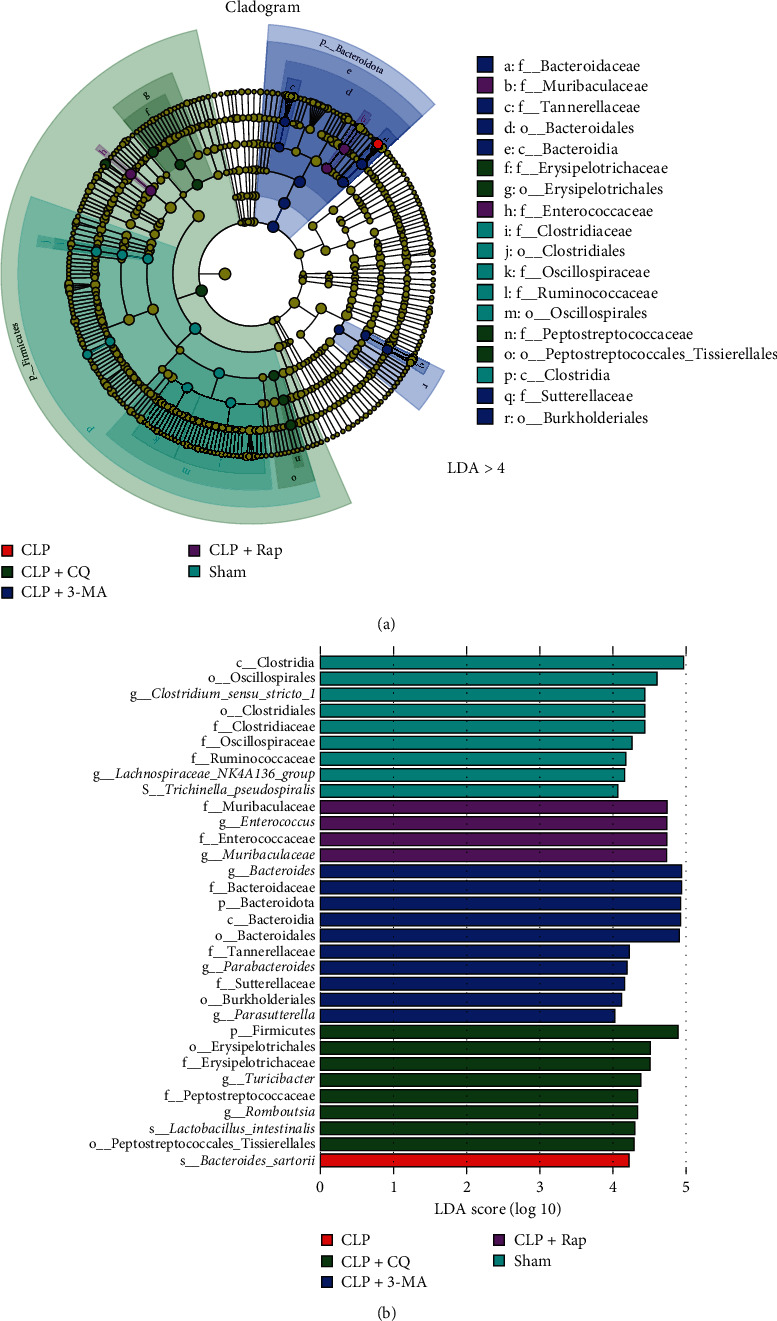
The protection of autophagy on sepsis altered the biomarkers in the microbial community. (A) Cladogram of ASVs (LDA > 4). The circle radiating from the inside to the outside represents the taxonomic level from phylum to species. Each small circle at different classification levels represents a classification at that level, and the diameter of the small circle is proportional to the relative abundance at that level. Coloring code: the biomarkers without significant differences are uniformly colored yellow, and the different biomarkers follow the group for coloring. (B) Barplot of ASVs (LDA > 4). ASV, amplicon sequence variants.

We further used LEfSe to analyze organisms with significant differences between groups. As shown in Figure 5A, LEfSe analysis revealed 32 distinguishing components at different taxon levels with an LDA score >4.0, including nine enriched in the sham group, four enriched in the Rap group, 10 enriched in the CLP + 3-MA group, eight enriched in the CLP + CQ group, and one enriched in the CLP group (*p* < 0.05, Figure 5). At the phylum level, the only one enriched in the CLP group belonged to Bacteroidetes, while the nine enriched in the sham group all belonged to Firmicutes. Interestingly, the four enriched in the CLP + Rap group belonged to Firmicutes (*n* = 2) and Bacteroidetes (*n* = 2), while the 18 enriched in the autophagy inhibition groups belonged to Firmicutes (*n* = 8), Bacteroidetes (*n* = 7), and Proteobacteria (*n* = 3; Figure 5B). Notably, most of the biomarkers of the CLP + 3-MA group were predominantly Proteobacteria (70%), while those of the CLP + CQ group were predominantly Firmicutes (100%; Figure 5B).

### 4.6. The Protection of Autophagy on Sepsis Influenced the Function of 16S rRNA Marker Genes Predicted Using Picrust2

**Figure 6:**
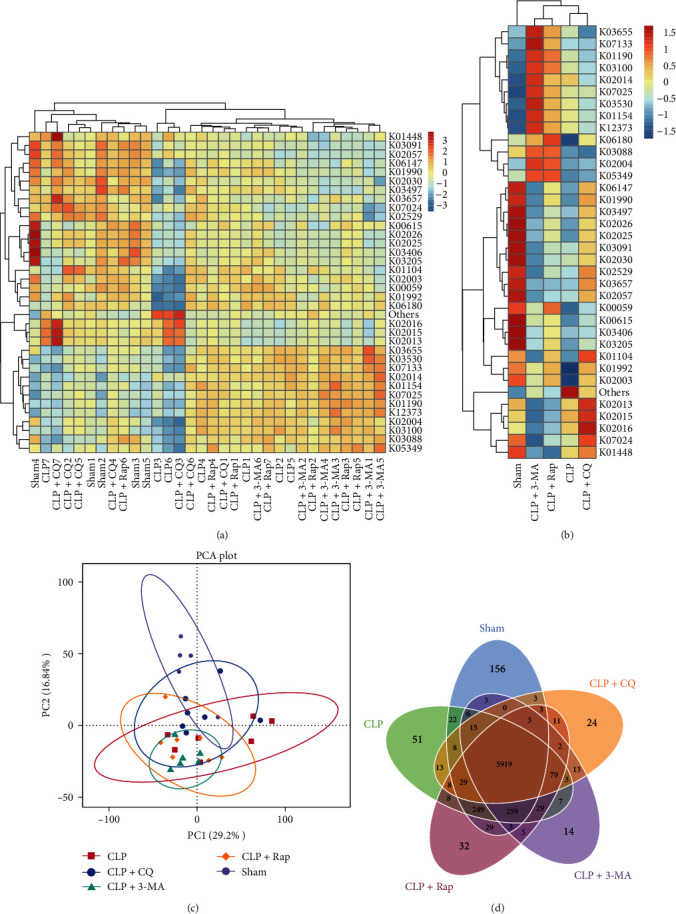
Functional predictions in the KEGG database using Picrust2. (A) Heatmap of the top 35 genes in each sample. (B) Heatmap of the top 35 genes in each group. (C) PCA of genes of the microbial communities in each group. (D) Venn diagram of genes in each group. KEGG, Kyoto encyclopedia of genes and genomes; KO: KEGG orthology; PCA, principal component analysis.

The top 35 functions in each sample were selected to draw a heatmap and cluster from different functional levels. Picrust2 is used for functional prediction in different databases, including clusters of orthologous gene (COG), protein family database (PFAM), Kyoto encyclopedia of genes and genomes (KEGG), TIGR families of aligned models (TIGRFAM), European commission (EC), and KEGG orthology (KO). This paper only shows the analysis results of the KO database (Figure 6). A heatmap of the top 35 functions in each sample or group is shown in Figure 6A,B. Compared with the sham group, 18 genes were significantly decreased after modeling but increased after autophagy activation, including K06180, K03088, K06147, K01990, K03497, K02026, K02025, K00059, K00615, K03406, K03205, K01104, K01992, and K02003 (Figure 6B). Most of these 18 genes still showed an upward trend after autophagy inhibition, but only K03088 showed a downward trend (Figure 6B). The functions of these 18 genes included RNA synthesis, ATP binding and transport, chromosome assignment, osmotic polysaccharide transport, transcytosis, and methylation.

## 5. Discussion

In previous studies, the positive intervention effect of autophagy on sepsis has been confirmed, but the specific mechanism is still unclear [8, 27]. In this study, we used autophagy activators or inhibitors to directly affect the level of autophagy and explored the potential mechanism by which autophagy protects against the inflammatory response and further changes the intestinal microbiota in septic rats.

Sepsis is a serious disease caused by life-threatening systemic infection. As an evolutionarily conserved degradation system, autophagy has long been recognized as a cellular adaptive protective mechanism in sepsis [28]. Autophagy modulation appears to be protective against multiple organ injuries in these murine sepsis models [8]. In this study, we used the autophagy enhancer Rap and the autophagy inhibitors 3-MA and CQ to change the autophagy level in septic rats to explore the protective mechanism of autophagy against sepsis. The results showed that autophagy regulators could regulate the level of autophagy and autophagic flux effectively, as detected by LC3-II, Beclin 1, and p62 (Figure 1). Interestingly, the autophagic inducer significantly decreased the mortality to 60% at 72 h and significantly decreased LPS levels in serum compared with the CLP group, while autophagic inhibitors showed the opposite effect (Figure 2). Furthermore, proinflammatory cytokines (TNF-*α*, IL-6, and IL-1*β*) were markedly improved, and the anti-inflammatory factor IL-10 increased significantly in the CLP + Rap group compared with the CLP group (Figure 2). Histopathological sections showed that multiple organs were severely damaged in sepsis model rats, and the activation of autophagy could effectively alleviate this pathological damage (Figures 2 and Supporting Information 1: Figure S1). These results indicated that autophagy was able to effectively protect septic rats by reducing inflammation and alleviating histopathological damage, which was completely consistent with previous studies [28, 29].

In addition to removing proteins to be degraded and damaged organelles, autophagy can also effectively remove bacteria and pathogens present in the cytoplasm [28]. Thus, whether changes in autophagy can alter the composition of the intestinal microbiota is an attractive topic. At present, increasing evidence shows that the intestinal microbiota plays a nonnegligible role in the formation and treatment of sepsis [30, 31]. In sepsis, many factors lead to inflammatory reactions, such as the destruction of mucosal barriers, intestinal microbiota disorders, use of antibiotics, and lack of intestinal nutrients, which in turn affect the metabolism and immunity of the host [32]. The gut microbiota plays an important role in the mortality rate and the immune response of septic animals, and it has now become a potential therapeutic target [33]. However, whether the changes in the intestinal microbiota in septic rats are related to the aforementioned autophagy or whether the protective effect of autophagy is achieved by regulating the intestinal microbiota remains unclear. To further evaluate the effects of autophagy on the gut microbiome, we collected the feces of experimental animals for 16S rRNA analysis. The results showed that the gut microbial richness and alpha diversity of the intestinal microbiota decreased significantly in septic rats, and activation of autophagy substantially protected the balance of the intestinal microbiota. This result coincides with the conclusion in many previous studies that drugs can improve inflammation and intestinal injury in sepsis by affecting autophagy and the intestinal microbiota [31, 34].

The gut microbiome affects the host's susceptibility and response to sepsis through multiple pathways [13]. To further investigate the protective and regulatory effects of autophagy on the gut microbiome, we further analyzed the structure of the intestinal bacteria with the highest abundances in each sample or group at each classification level. The results showed that the abundances of Proteobacteria, Myxococcota, and Verrucomicrobiota were significantly increased in the CLP group compared with the sham group, whereas those of Firmicutes and Bacteroidetes were significantly decreased. Autophagy activation and protection on sepsis significantly changed the abundances of Proteobacteria and Firmicutes, while autophagy inhibitors, especially the inhibition of autophagic flux, had no such effect (Figure 3). Proteobacteria is a common factor in human diseases [35]. The subpopulation of Proteobacteria with adhesive and invasive ability may utilize genetic defects in pathogen recognition and bacterial clearance, allowing them to proliferate uncontrollably and providing a trigger for inflammation [36]. Our experiments found that the induction of autophagy in sepsis markedly reduced the abundance of Proteobacteria (Figure 3). This result suggested that there was a close relationship between autophagy, the intestinal microbiota, and sepsis inflammation, which deserves further study. The clustering results showed that the abundance of *Acidobacteriota* and other phyla with higher abundances in the sham group decreased significantly in the CLP group, while Proteobacteria, Myxococcota, and Verrucomicrobiota with lower abundances increased significantly in the CLP group. Desulfobacterota and other phyla increased significantly after autophagy induction, but alterations in response to autophagy inhibition did not occur (Figure 3). These results are similar to some previous results in various inflammation-related diseases [6, 37].

At the genus level, we found the abundance of *Bacteroides* and *Escherichia–Shigella* was increased in septic rats, while that of Muribaculaceae and *Prevotella* was decreased. While autophagy was activated, the abundance of these bacteria reversed (Figure 3). At present, there are few relevant reports on the relationship between autophagy and these microorganisms, but we can understand their links with disease and health in many studies. *Bacteroides* is a common pathogenic bacterium in sepsis and also belongs to opportunistic pathogens, which may cause endogenous infections. *Escherichia–Shigella* is believed to be associated with longevity and nonalcoholic fatty liver disease. The relative abundance of *Escherichia–Shigella* in patients with nonalcoholic fatty liver disease is significantly higher than that in the healthy population group [38]. Long-lived individuals exhibit a unique intestinal pattern characterized by the coexistence of Bacteroidetes and *Escherichia–Shigella* genera, which are dominant in young adults and other elderly populations, respectively [39]. Prevotella has the function of immune modification and inhibition of inflammatory factors. In inflammatory diseases, the *Escherichia* and *Bacteroides* genes are generally increased, while *Prevotella* is almost depleted [40]. The above researches are similar to our results. Autophagy activation improved the abundance of beneficial bacteria and reduced the number of harmful bacteria in septic rats to some extent.

Based on the comprehensive analysis of difference significance and LEfSe (LDA > 4), we identified biomarkers between groups in the microbial community (Figure 5 and Supporting Information 1: Figure S4). We found that the abundances of Muribaculaceae and *Ruminococcus* decreased significantly after modeling but recovered significantly after autophagy-induced intervention. The nutritional relationship between microorganisms includes cooperation and competition. Ruminococcaceae is an important family of Firmicutes bacteria that have evolved specialized systems to utilize complex carbohydrates [41]. It is able to decompose resistant starch and release energy, thus providing nutrition for the intestinal microbiota and short-chain fatty acids (SCFAs) for the host [42]. Obviously, the systemic infection caused by CLP affected the abundance of this important microbiota, while the protective effect of autophagy restored the abundance of Ruminococcaceae to a certain extent. Some intestinal microbiota can compete with pathogens for mucus-derived sugar as their key nutritional source. Therefore, they are ecological gatekeepers of healthy intestines and attractive candidates for the treatment of infectious diseases [43]. Muribaculaceae, an underexplored family, is the major mucin monosaccharide family. They impede *Clostridioides difficile*'s access to mucosal sugars and impair pathogen colonization in antibiotic-treated mice [43]. In our studies, the abundance of Murbaculaceae significantly decreased after modeling, which significantly weakened nutritional competition, thus accelerating the colonization of pathogenic bacteria in the intestine. Autophagy protection significantly increased the abundance of Muribaculaceae and delayed the process of sepsis. In conclusion, our results can be well explained using previous studies, but the specific mechanism needs further study.

Then, how did the autophagy protection to sepsis alter the gut microbiome? Yan et al. [44] demonstrated that defects in autophagy in the hepatocytes caused changes in bile acid levels and composition, which in turn affected the gut microbiome. Another study showed that the microbiome regulated energy metabolism and autophagy [45]. According to the functional annotation and abundance information in each sample, we further drew a heatmap and cluster from different functional levels using the top 35 functions (Figure 6). We found 18 genes that significantly decreased after modeling but increased postautophagy activation, including RNA synthesis, ATP binding and transport, chromosome assignment, osmotic polysaccharide transport, transketosis, and methylation (Figure 6). These changes indicate that autophagy can regulate the intestinal microbiota in many ways, but further experimental analysis is needed on the specific regulatory mechanism.

In conclusion, our research suggests that autophagy can improve inflammation and directly or indirectly regulate the intestinal microbiota in septic rats, which plays a crucial role in survival and represents a potential target for therapeutic intervention. However, there are still some shortcomings in our research. Sepsis causes inflammatory storm, autophagy inhibition, and changes of gut microbiota. Through our experiments, we can only prove that the activation of autophagy can improve the inflammatory response, while it is still unknown whether changes in gut microbiota are directly caused by changes in autophagy or by the alleviation of inflammation in animals themselves. In addition, autophagy is a double-edged sword, and the relationship between its level and multibacterial infection immunity is not yet clear. In all, more experiments are needed to explain the relationship between them and elucidate the specific molecular mechanisms.

## Figures and Tables

**Table 1:** Alpha diversity in different groups.

Variable	Sham	CLP	CLP		
			Rap	3-MA	CQ
Reads	107,529 ± 4845	105,675 ± 4179	109,216 ± 6504	106,353 ± 3256	110,279 ± 5036
Chao1	819.87 ± 111.01	548.70 ± 32.40^a^	604.50 ± 51.56^a^	572.06 ± 36.58^a^	595.55 ± 65.00^a^
Shannon	7.11 ± 0.28	6.08 ± 0.50^a^	6.69 ± 0.35^b^	6.64 ± 0.29 ^ab^	6.27 ± 0.66 ^a^
Simpson	0.97 ± 0.01	0.95 ± 0.02^a^	0.96 ± 0.02	0.97 ± 0.01^b^	0.95 ± 0.03
Observed-ASVs	819.60 ± 123.90	547.43 ± 33.08	603.29 ± 51.58	571.67 ± 36.78	593.43 ± 66.02

*Note:* Kruskal‒Wallis test, false discovery rate <0.05.

Abbreviations: 3-MA, 3-methyladenine; ASVs, amplicon sequence variants; CLP, cecum ligation perforation; CQ, chloroquine; Rap, rapamycin.

^a^Versus the sham group.

^b^Versus the CLP group.

## Data Availability

The data can be requested from the author upon reasonable request.

## Nomenclature

SD: Sprague‒Dawley
CLP: Cecum ligation perforation
Rap: Rapamycin
3-MA: 3-methyladenosine
HE: Histopathological examination
LPS: Lipopolysaccharide
TNF-*α*: Tumor necrosis factor-*α*
IL-6: Interleukin-6
IL-1*β*: Interleukin-1*β*
IL-10: Interleukin-10
MODS: Multiple organ dysfunction syndrome
IBD: Inflammatory bowel disease
ATG: Autophagy-related protein
ELISA: Enzyme-linked immunosorbent assay
GAPDH: Glyceraldehyde 3 phosphate dehydrogenase
PCA: Principal component analysis
PCoA: Principal coordinate analysis
ASV: Amplicon sequence variants
LEfSe: LDA effect size.

## Supporting Information

Supporting Information 1 Figure S1: Histological appearance in the five groups after H&E staining (upper panel ×100 and lower panel ×200). (A) Liver. (B) Heart. (C) Spleen. (D) Kidney. Figure S2: Alpha diversity indices and various rarefaction curves. (A) ASVs observed in the feces of the five groups. (B) Dominance of the gut microbiota in the five groups. (C) Pielou's evenness index of the gut microbiota in the five groups. (D–F) Chao1, Shannon, and Simpson index comparisons of the gut microbiota in the five groups. Figure S3: Top 10 taxa with the highest abundances in each sample or group at each classification level (class, order, family, and species). Figure S4: Analysis of significance between groups at the genus level using a *t* test.

Supporting Information 2 Table S1: *t* test analysis at genus level.

Supporting Information 3 Table S2: Metastat analysis at genus level.
