# Supplementary material for: Autophagy Improves Inflammatory Response in Sepsis Accompanied by Changes in Gut Microbiota
Source: Mediators Inflamm. 2024 Oct 18;2024:9550301. doi: 10.1155/2024/9550301 (PMC11511597; doi:10.1155/2024/9550301)
Supplement: Supporting Information 1 — Figure S1: Histological appearance in the five groups after H&E staining (upper panel ×100 and lower panel ×200). (A) Liver. (B) Heart. (C) Spleen. (D) Kidney. Figure S2: Alpha diversity indices and various rarefaction curves. (A) ASVs observed in the feces of the five groups. (B) Dominance of the gut microbiota in the five groups. (C) Pielou's evenness index of the gut microbiota in the five groups. (D–F) Chao1, Shannon, and Simpson index comparisons of the gut microbiota in the five groups. Figure S3: Top 10 taxa with the highest abundances in each sample or group at each classification level (class, order, family, and species). Figure S4: Analysis of significance between groups at the genus level using a t test. [file 9550301.f1.doc]

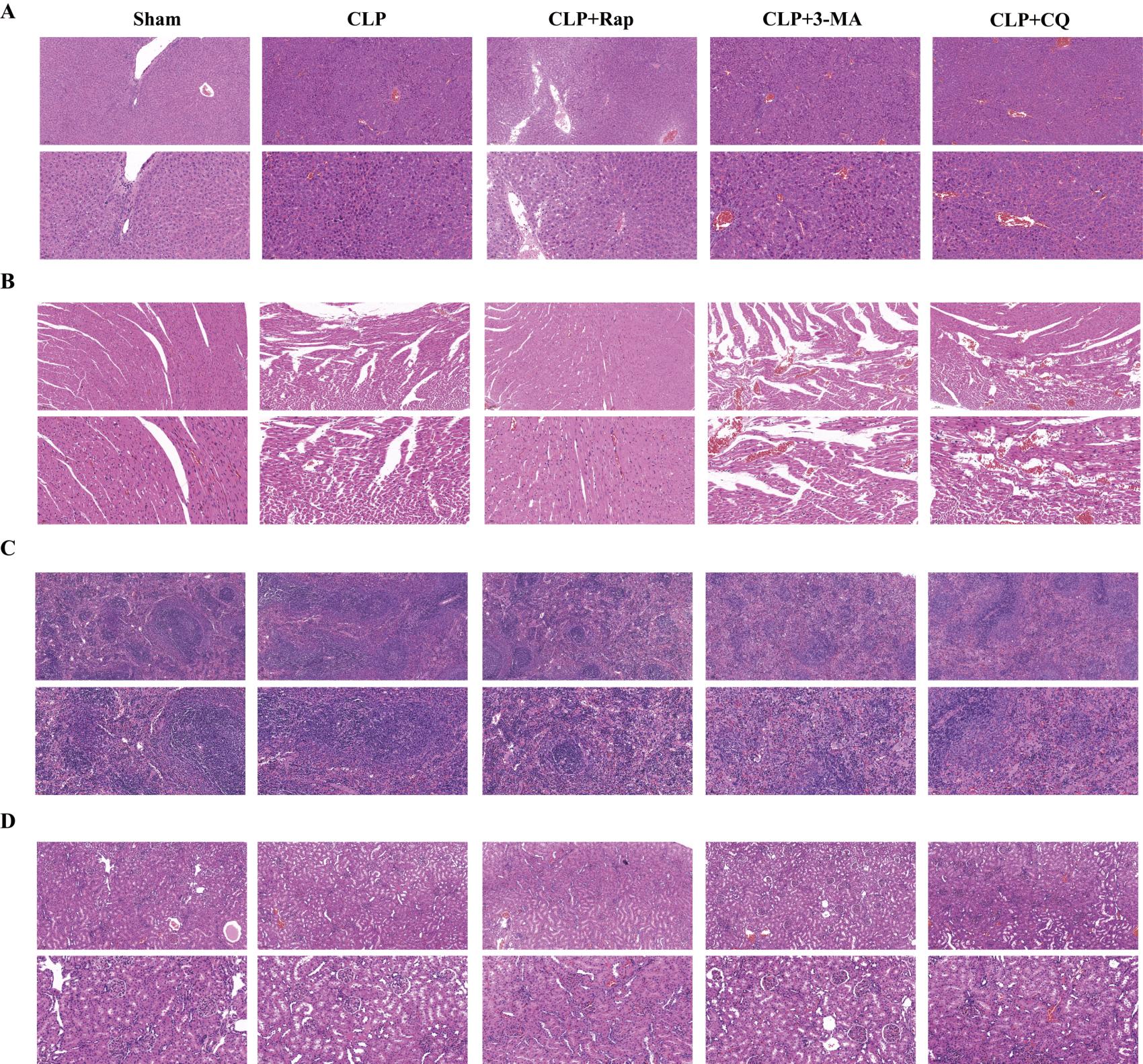


Figure S1 Histological appearance in the five groups after H&E staining.


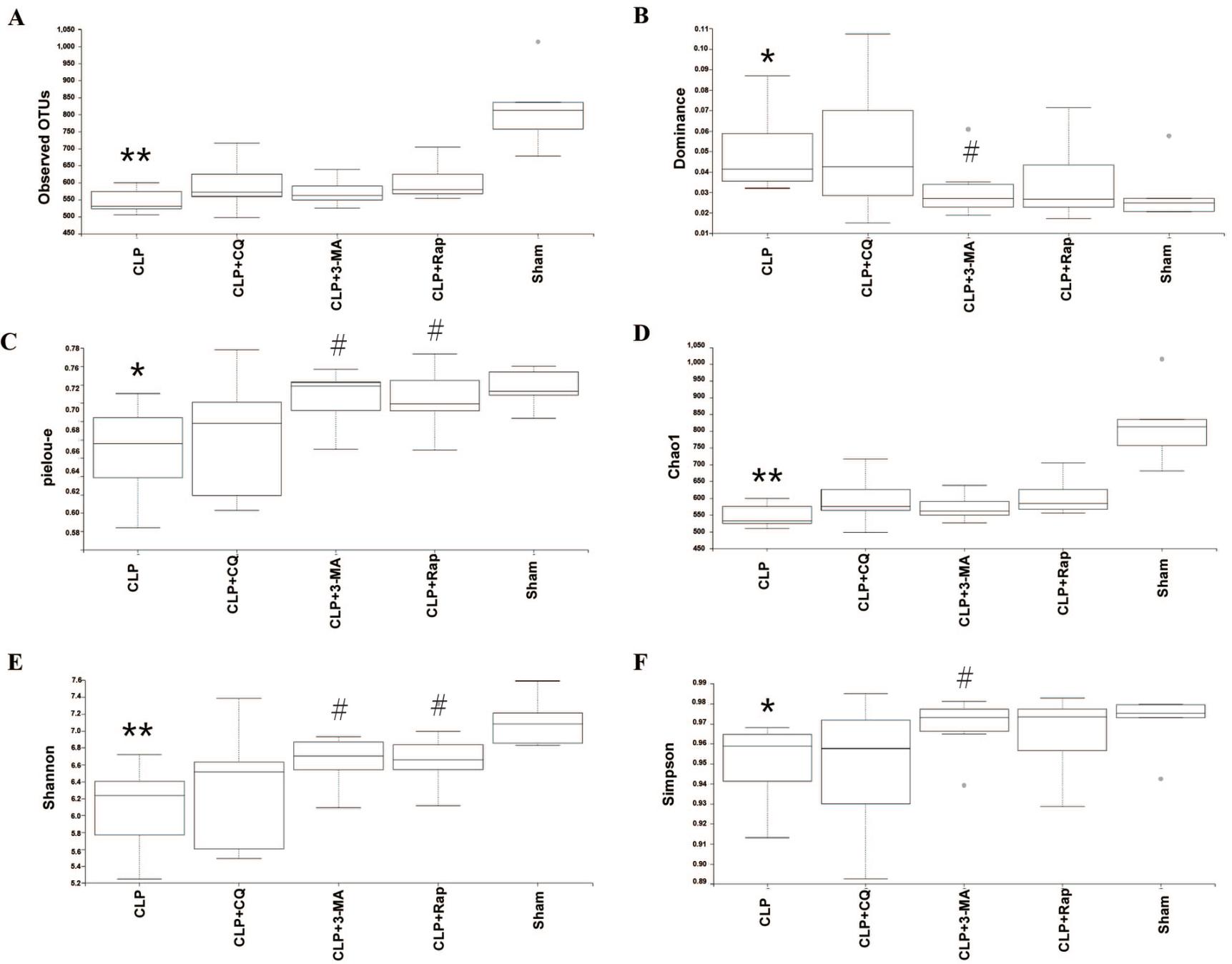


Figure S2 Alpha diversity indices and various rarefaction curves.


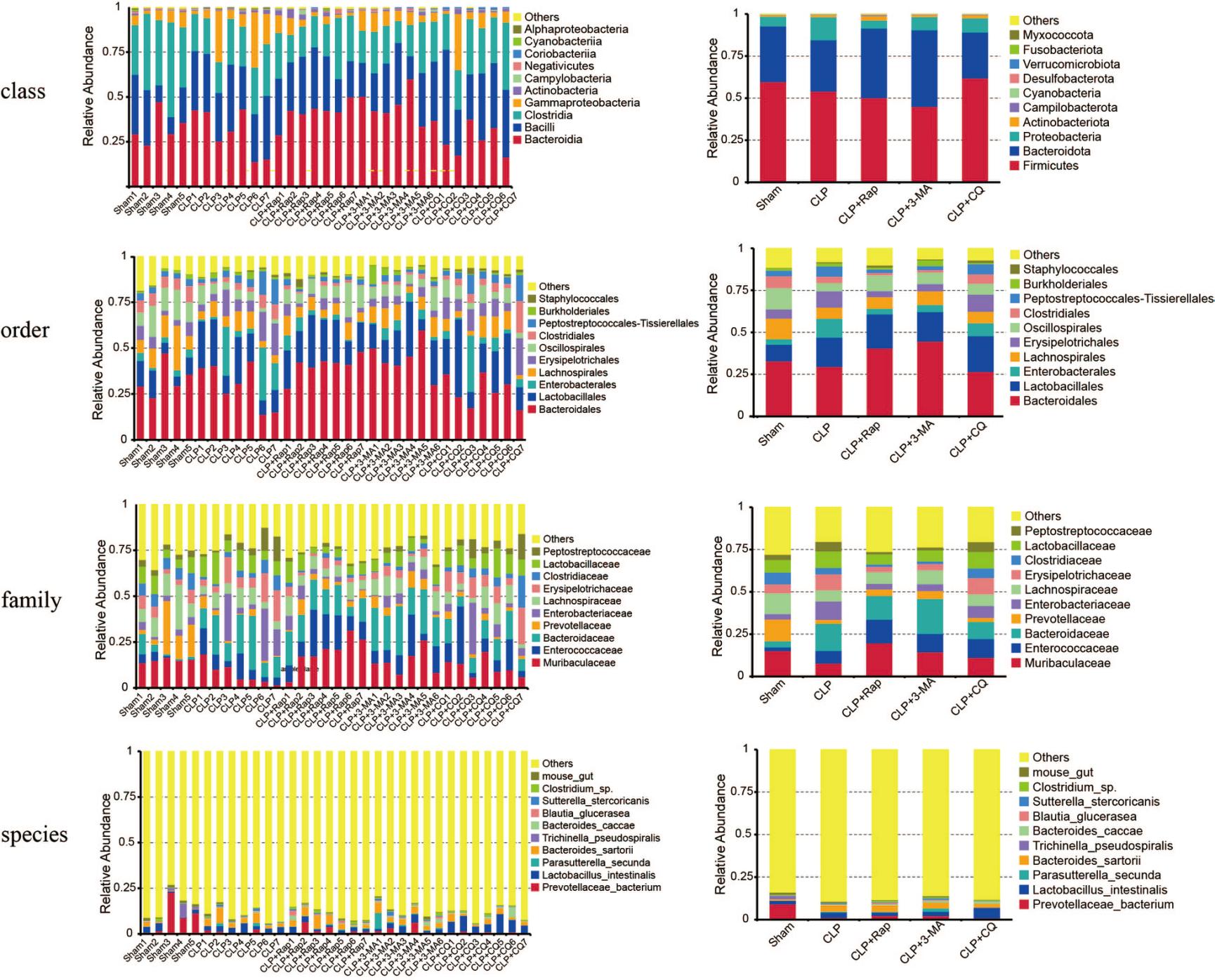


Figure S3 Top 10 taxa with the highest abundances in each sample or group at each classification level (class, order, family, and species).


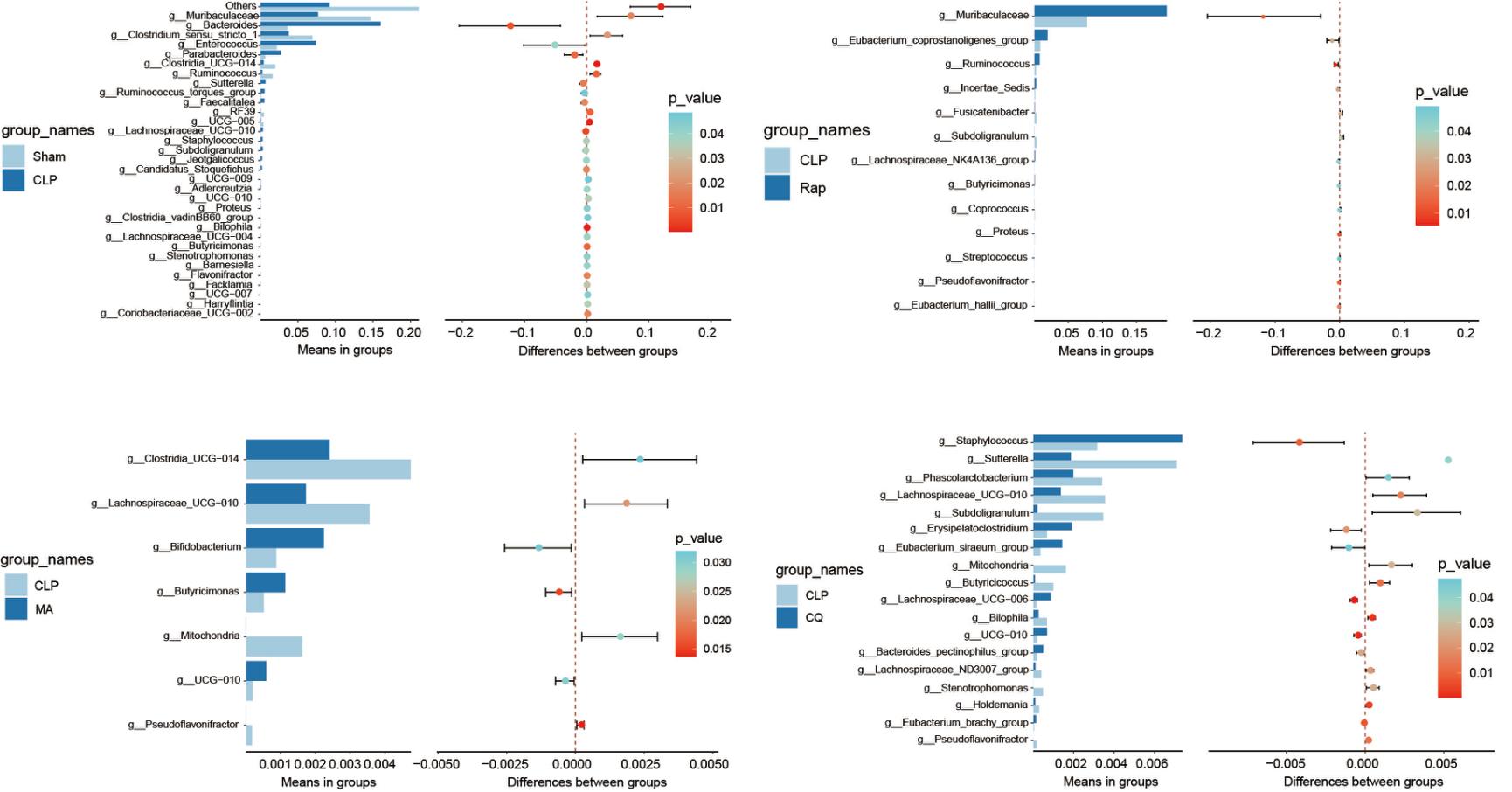


Figure S4 Analysis of significance between groups at the genus level using a t test.
