# Supplementary material for: Autophagy Improves Inflammatory Response in Sepsis Accompanied by Changes in Gut Microbiota
Source: Mediators Inflamm. 2024 Oct 18;2024:9550301. doi: 10.1155/2024/9550301 (PMC11511597; doi:10.1155/2024/9550301)
Supplement: Supporting Information 3 — Table S2: Metastat analysis at genus level. [file 9550301.f3.pdf]

Table S2 Metastat analysis at genus level
Sham vs CLP
k__Bacteria;p__Bacteroidota;c__Bacteroidia;o__Bacteroidales;f__Muribaculaceae;g__Muribaculaceae;
k__Bacteria;p__Bacteroidota;c__Bacteroidia;o__Bacteroidales;f__Bacteroidaceae;g__Bacteroides;
k__Bacteria;p__Firmicutes;c__Bacilli;o__Lactobacillales;f__Enterococcaceae;g__Enterococcus;
k__Bacteria;p__Firmicutes;c__Clostridia;o__Clostridiales;f__Clostridiaceae;g__Clostridium_sensu_stricto_1;
k__Bacteria;p__Bacteroidota;c__Bacteroidia;o__Bacteroidales;f__Tannerellaceae;g__Parabacteroides;
k__Bacteria;p__Firmicutes;c__Clostridia;o__Oscillospirales;f__Ruminococcaceae;g__Ruminococcus;
k__Bacteria;p__Firmicutes;c__Clostridia;o__Lachnospirales;f__Lachnospiraceae;g__Ruminococcus_torques_
k__Bacteria;p__Firmicutes;c__Clostridia;o__Clostridia_UCG-014;f__Clostridia_UCG-014;g__Clostridia_UC
k__Bacteria;p__Firmicutes;c__Bacilli;o__Staphylococcales;f__Staphylococcaceae;g__Jeotgalicoccus;
k__Bacteria;p__Firmicutes;c__Bacilli;o__Staphylococcales;f__Staphylococcaceae;g__Staphylococcus;
k__Bacteria;p__Firmicutes;c__Clostridia;o__Lachnospirales;f__Lachnospiraceae;g__Roseburia;
k__Bacteria;p__Proteobacteria;c__Gammaproteobacteria;o__Burkholderiales;f__Sutterellaceae;g__Sutterella;
k__Bacteria;p__Proteobacteria;c__Gammaproteobacteria;o__Aeromonadales;f__Succinivibrionaceae;g__Succi
k__Bacteria;p__Firmicutes;c__Clostridia;o__Lachnospirales;f__Lachnospiraceae;g__Lachnospiraceae_UCG-0
k__Bacteria;p__Firmicutes;c__Bacilli;o__Erysipelotrichales;f__Erysipelotrichaceae;g__Faecalitalea;
k__Bacteria;p__Firmicutes;c__Bacilli;o__Bacillales;f__Bacillaceae;g__Bacillus;
k__Bacteria;p__Firmicutes;c__Clostridia;o__Lachnospirales;f__Lachnospiraceae;g__Eubacterium_xylanophil
k__Bacteria;p__Firmicutes;c__Clostridia;o__Oscillospirales;f__Ruminococcaceae;g__Subdoligranulum;
k__Bacteria;p__Actinobacteriota;c__Actinobacteria;o__Propionibacteriales;f__Propionibacteriaceae;g__Cutib
k__Bacteria;p__Firmicutes;c__Bacilli;o__RF39;f__RF39;g__RF39;
k__Bacteria;p__Proteobacteria;c__Gammaproteobacteria;o__Pseudomonadales;f__Moraxellaceae;g__Enhydro
k__Bacteria;p__Proteobacteria;c__Gammaproteobacteria;o__Pseudomonadales;f__Pseudomonadaceae;g__Pse
k__Bacteria;p__Firmicutes;c__Bacilli;o__Erysipelotrichales;f__Erysipelatoclostridiaceae;g__Candidatus_Stoq
k__Bacteria;p__Firmicutes;c__Clostridia;o__Oscillospirales;f__Ruminococcaceae;g__Incertae_Sedis;
k__Bacteria;p__Actinobacteriota;c__Coriobacteriia;o__Coriobacteriales;f__Eggerthellaceae;g__Adlercreutzia;
k__Bacteria;p__Firmicutes;c__Clostridia;o__Oscillospirales;f__Oscillospiraceae;g__UCG-005;
k__Bacteria;p__Firmicutes;c__Bacilli;o__Lactobacillales;f__Streptococcaceae;g__Streptococcus;
k__Bacteria;p__Bacteroidota;c__Bacteroidia;o__Bacteroidales;f__Prevotellaceae;g__Prevotellaceae_UCG-00
k__Bacteria;p__Firmicutes;c__Negativicutes;o__Veillonellales-Selenomonadales;f__Selenomonadaceae;g__A
k__Bacteria;p__Bacteroidota;c__Bacteroidia;o__Bacteroidales;f__Marinifilaceae;g__Butyricimonas;
k__Bacteria;p__Proteobacteria;c__Gammaproteobacteria;o__Enterobacterales;f__Morganellaceae;g__Proteus;
k__Bacteria;p__Firmicutes;c__Negativicutes;o__Veillonellales-Selenomonadales;f__Selenomonadaceae;g__Q
k__Bacteria;p__Firmicutes;c__Clostridia;o__Oscillospirales;f__Ruminococcaceae;g__CAG-352;
k__Bacteria;p__Firmicutes;c__Clostridia;o__Monoglobales;f__Monoglobaceae;g__Monoglobus;
k__Bacteria;p__Firmicutes;c__Clostridia;o__Oscillospirales;f__Butyricicoccaceae;g__UCG-009;
k__Bacteria;p__Bacteroidota;c__Bacteroidia;o__Bacteroidales;f__Rikenellaceae;g__Rikenellaceae_RC9_gut_
k__Bacteria;p__Firmicutes;c__Clostridia;o__Oscillospirales;f__UCG-010;g__UCG-010;
k__Bacteria;p__Firmicutes;c__Bacilli;o__Lactobacillales;f__Carnobacteriaceae;g__Atopostipes;
k__Bacteria;p__Firmicutes;c__Bacilli;o__Lactobacillales;f__Aerococcaceae;g__Facklamia;
k__Bacteria;p__Firmicutes;c__Bacilli;o__Erysipelotrichales;f__Erysipelatoclostridiaceae;g__Erysipelotrichace
k__Bacteria;p__Firmicutes;c__Clostridia;o__Lachnospirales;f__Lachnospiraceae;g__Ruminococcus_gnavus_g
k__Bacteria;p__Firmicutes;c__Clostridia;o__Oscillospirales;f__Ruminococcaceae;g__Faecalibacterium;
k__Bacteria;p__Firmicutes;c__Clostridia;o__Lachnospirales;f__Lachnospiraceae;g__Lachnospiraceae_UCG-0
k__Bacteria;p__Firmicutes;c__Clostridia;o__Oscillospirales;f__Oscillospiraceae;g__Flavonifractor;
k__Bacteria;p__Bacteroidota;c__Bacteroidia;o__Bacteroidales;f__Barnesiellaceae;g__Barnesiella;
k__Bacteria;p__Firmicutes;c__Bacilli;o__Bacillales;f__Planococcaceae;g__Rummeliibacillus;
k__Bacteria;p__Firmicutes;c__Clostridia;o__Lachnospirales;f__Lachnospiraceae;g__Sellimonas;
k__Bacteria;p__Firmicutes;c__Clostridia;o__Lachnospirales;f__Lachnospiraceae;g__Lachnospiraceae_UCG-0
k__Bacteria;p__Firmicutes;c__Clostridia;o__Lachnospirales;f__Lachnospiraceae;g__Acetitomaculum;
k__Bacteria;p__Spirochaetota;c__Spirochaetia;o__Spirochaetales;f__Spirochaetaceae;g__Sphaerochaeta;
k__Bacteria;p__Proteobacteria;c__Gammaproteobacteria;o__Xanthomonadales;f__Xanthomonadaceae;g__Ste
k__Bacteria;p__Firmicutes;c__Clostridia;o__Peptostreptococcales-Tissierellales;f__Anaerovoracaceae;g__Eub
k__Bacteria;p__Actinobacteriota;c__Coriobacteriia;o__Coriobacteriales;f__Atopobiaceae;g__Coriobacteriace
k__Bacteria;p__Firmicutes;c__Bacilli;o__Staphylococcales;f__Staphylococcaceae;g__Salinicoccus;

k__Bacteria;p__Firmicutes;c__Clostridia;o__Lachnospirales;f__Lachnospiraceae;g__Eubacterium_ruminantiu
k__Bacteria;p__Firmicutes;c__Clostridia;o__Clostridia_vadinBB60_group;f__Clostridia_vadinBB60_group;g_
k__Bacteria;p__Desulfobacterota;c__Desulfovibrionia;o__Desulfovibrionales;f__Desulfovibrionaceae;g__Bilo
k__Bacteria;p__Firmicutes;c__Bacilli;o__Bacillales;f__Planococcaceae;g__Sporosarcina;
k__Bacteria;p__Firmicutes;c__Clostridia;o__Oscillospirales;f__Oscillospiraceae;g__UCG-007;
k__Bacteria;p__Firmicutes;c__Bacilli;o__Lactobacillales;f__Aerococcaceae;g__Globicatella;
k__Bacteria;p__Actinobacteriota;c__Coriobacteriia;o__Coriobacteriales;f__Coriobacteriaceae;g__Collinsella;
k__Bacteria;p__Firmicutes;c__Clostridia;o__Peptococcales;f__Peptococcaceae;g__Peptococcus;
k__Bacteria;p__Verrucomicrobiota;c__Kiritimatiellae;o__WCHB1-41;f__WCHB1-41;g__WCHB1-41;
k__Bacteria;p__Firmicutes;c__Clostridia;o__Lachnospirales;f__Lachnospiraceae;g__Oribacterium;
k__Bacteria;p__Bacteroidota;c__Bacteroidia;o__Bacteroidales;f__Rikenellaceae;g__Rikenella;
k__Bacteria;p__Firmicutes;c__Clostridia;o__Oscillospirales;f__Ruminococcaceae;g__Harryflintia;
k__Bacteria;p__Spirochaetota;c__Spirochaetia;o__Spirochaetales;f__Spirochaetaceae;g__Treponema;
k__Bacteria;p__Firmicutes;c__Bacilli;o__Lactobacillales;f__Aerococcaceae;g__Aerococcus;
k__Bacteria;p__Firmicutes;c__Clostridia;o__Lachnospirales;f__Lachnospiraceae;g__ASF356;
k__Bacteria;p__Proteobacteria;c__Gammaproteobacteria;o__Burkholderiales;f__Oxalobacteraceae;g__Hermin
k__Bacteria;p__Proteobacteria;c__Gammaproteobacteria;o__Burkholderiales;f__Burkholderiaceae;g__Burkho
k__Bacteria;p__Proteobacteria;c__Gammaproteobacteria;o__Aeromonadales;f__Succinivibrionaceae;g__Anae
k__Bacteria;p__Elusimicrobiota;c__Elusimicrobia;o__Elusimicrobiales;f__Elusimicrobiaceae;g__Elusimicrobi
k__Bacteria;p__Bacteroidota;c__Bacteroidia;o__Bacteroidales;f__Prevotellaceae;g__Prevotellaceae_Ga6A1_g
k__Bacteria;p__Proteobacteria;c__Gammaproteobacteria;o__Burkholderiales;f__Alcaligenaceae;g__Alcaligen
k__Bacteria;p__Firmicutes;c__Bacilli;o__Lactobacillales;f__Carnobacteriaceae;g__Carnobacterium;
k__Bacteria;p__Firmicutes;c__Bacilli;o__Lactobacillales;f__Leuconostocaceae;g__Weissella;
k__Bacteria;p__Firmicutes;c__Clostridia;o__Lachnospirales;f__Lachnospiraceae;g__Eubacterium_ventriosum
k__Bacteria;p__Proteobacteria;c__Gammaproteobacteria;o__Burkholderiales;f__Oxalobacteraceae;g__Janthin
k__Bacteria;p__Firmicutes;c__Bacilli;o__Erysipelotrichales;f__Erysipelotrichaceae;g__Catenisphaera;
k__Bacteria;p__Firmicutes;c__Clostridia;o__Peptostreptococcales-Tissierellales;f__Peptostreptococcaceae;g__
k__Bacteria;p__Bacteroidota;c__Bacteroidia;o__Bacteroidales;f__p-2534-18B5_gut_group;g__p-2534-18B5_g
k__Bacteria;p__Patescibacteria;c__Saccharimonadia;o__Saccharimonadales;f__Saccharimonadaceae;g__Cand
k__Bacteria;p__Firmicutes;c__Clostridia;o__Lachnospirales;f__Lachnospiraceae;g__GCA-900066755;
k__Bacteria;p__Bacteroidota;c__Bacteroidia;o__Bacteroidales;f__Bacteroidales_RF16_group;g__Bacteroidale
k__Bacteria;p__Actinobacteriota;c__Coriobacteriia;o__Coriobacteriales;f__Atopobiaceae;g__Olsenella;
k__Bacteria;p__Fibrobacterota;c__Fibrobacteria;o__Fibrobacterales;f__Fibrobacteraceae;g__Fibrobacter;
k__Bacteria;p__Firmicutes;c__Negativicutes;o__Veillonellales-Selenomonadales;f__Veillonellaceae;g__Mega
k__Bacteria;p__Acidobacteriota;c__Vicinamibacteria;o__Vicinamibacterales;f__Vicinamibacteraceae;g__Vici
k__Bacteria;p__Proteobacteria;c__Alphaproteobacteria;o__Caulobacterales;f__Caulobacteraceae;g__Caulobac
k__Bacteria;p__Acidobacteriota;c__Blastocatellia;o__Pyrinomonadales;f__Pyrinomonadaceae;g__RB41;
k__Bacteria;p__Actinobacteriota;c__Actinobacteria;o__Micromonosporales;f__Micromonosporaceae;g__Dact
k__Bacteria;p__Bacteroidota;c__Bacteroidia;o__Bacteroidales;f__Rikenellaceae;g__dgA-11_gut_group;
k__Bacteria;p__Myxococcota;c__Polyangia;o__Polyangiales;f__Phaselicystidaceae;g__Phaselicystis;
Others

p.value	CLP vs Rap	p.value
0.003364807	k__Bacteria;p__Bacteroidota;c__Bacteroidia;o__	0.004507463
0.002034335	k__Bacteria;p__Firmicutes;c__Bacilli;o__Lactoba	0.043199005
0.020742489	k__Bacteria;p__Firmicutes;c__Clostridia;o__Pept	0.027910448
0.007339056	k__Bacteria;p__Firmicutes;c__Clostridia;o__Clos	0.024442786
0.002901288	k__Bacteria;p__Firmicutes;c__Clostridia;o__Lacl	0.007850746
0.000334764	k__Bacteria;p__Firmicutes;c__Clostridia;o__Osc	0.010452736
0.027090129	k__Bacteria;p__Actinobacteriota;c__Actinobacte	0.018139303
0	k__Bacteria;p__Firmicutes;c__Clostridia;o__Lacl	0.047482587
0.01944206	k__Bacteria;p__Firmicutes;c__Clostridia;o__Lacl	0.035676617
0.016141631	k__Bacteria;p__Firmicutes;c__Clostridia;o__Osc	0.001024876
0.021154506	k__Bacteria;p__Firmicutes;c__Clostridia;o__Osc	0.007975124
0.002480687	k__Bacteria;p__Firmicutes;c__Clostridia;o__Osc	0.007318408
0.023103004	k__Bacteria;p__Proteobacteria;c__Gammaproteol	0.00120398
0.001257511	k__Bacteria;p__Firmicutes;c__Clostridia;o__Lacl	0.015855721
0.003613734	k__Bacteria;p__Bacteroidota;c__Bacteroidia;o__	0.022029851
0.035896996	k__Bacteria;p__Firmicutes;c__Clostridia;o__Clos	0.032905473
0.018360515	k__Bacteria;p__Firmicutes;c__Clostridia;o__Lacl	0.013089552
0.011502146	k__Bacteria;p__Firmicutes;c__Bacilli;o__Erysipe	0.029701493
0.045145923	k__Bacteria;p__Firmicutes;c__Clostridia;o__Lacl	0.044069652
0.000390558	k__Bacteria;p__Proteobacteria;c__Gammaproteol	0.032303483
0.012600858	k__Bacteria;p__Firmicutes;c__Clostridia;o__Osc	0.027820896
0.038236052	k__Bacteria;p__Firmicutes;c__Bacilli;o__Lactoba	0.016283582
0.005480687	k__Bacteria;p__Firmicutes;c__Bacilli;o__Lactoba	0.042482587
0.037321888	k__Bacteria;p__Firmicutes;c__Clostridia;o__Lacl	0.005452736
0.021493562	k__Bacteria;p__Firmicutes;c__Clostridia;o__Osc	0.001547264
5.15021E-05	k__Bacteria;p__Proteobacteria;c__Gammaproteol	0.030865672
0.047587983	k__Bacteria;p__Firmicutes;c__Bacilli;o__Lactoba	0.031249407
0.01795279	k__Bacteria;p__Firmicutes;c__Clostridia;o__Pept	0.000502488
0.018506438	k__Bacteria;p__Firmicutes;c__Bacilli;o__Lactoba	0.003189055
0.002892704	k__Bacteria;p__Proteobacteria;c__Gammaproteol	0.041781095
0.01483691	k__Bacteria;p__Proteobacteria;c__Alphaproteoba	0.035218905
0.024875536	k__Bacteria;p__Actinobacteriota;c__Actinobacte	0.049338308
0.018703863	k__Bacteria;p__Firmicutes;c__Clostridia;o__Pept	0.037358209
0.018343348	k__Bacteria;p__Verrucomicrobiota;c__Lentispha	0.031249407
0.008690987	k__Bacteria;p__Actinobacteriota;c__Actinobacte	0.031249407
0.019240343		
0.004334764		
0.033201717		
0.007536481		
0.036420601		
0.028909871		
0.022291845		
0.013622318		
0.005227468		
0.018463519		
0.046141631		
0.02288412		
0.011536481		
0.022038627		
0.023566524		
0.013918455		
0.024635193		
0.005454936		
0.028506438		

0.021390558
0.011515021
5.5794E-05
0.025167382
0.007042918
0.042682403
0.025626609
0.026828326
0.03060515
0.031476395
0.048454936
0.004909871
0.032824034
0.023888412
0.037017167
0.009832618
0.016991416
0.026416309
0.035922747
0.018995708
0.015214592
0.010527897
0.00044206
0.021206009
0.015536481
0.020806867
0.029695279
0.017476395
0.020519313
0.04463241
0.031982833
0.04463241
0.020012876
0.030140445
0.030140445
0.030140445
0.030140445
0.030140445
0.030077253
0.030140445
8.15451E-05

CLP vs MA	p.value	CLP vs CQ
k__Bacteria;p__Actinobacteriota;c__	0.014313514	k__Bacteria;p__Proteobacteria;c__Gam
k__Bacteria;p__Firmicutes;c__Negati	0.043810811	k__Bacteria;p__Proteobacteria;c__Gam
k__Bacteria;p__Firmicutes;c__Clostr	0.010778378	k__Bacteria;p__Firmicutes;c__Bacilli;o_
k__Bacteria;p__Firmicutes;c__Clostr	0.01	k__Bacteria;p__Firmicutes;c__Clostridi
k__Bacteria;p__Cyanobacteria;c__Cy	0.047383784	k__Bacteria;p__Firmicutes;c__Negativi
k__Bacteria;p__Proteobacteria;c__Ga	0.011178378	k__Bacteria;p__Firmicutes;c__Clostridi
k__Bacteria;p__Proteobacteria;c__Al	0.006448649	k__Bacteria;p__Firmicutes;c__Clostridi
k__Bacteria;p__Bacteroidota;c__Bac	0.004589189	k__Bacteria;p__Firmicutes;c__Clostridi
k__Bacteria;p__Firmicutes;c__Clostr	0.034037838	k__Bacteria;p__Cyanobacteria;c__Cyan
k__Bacteria;p__Firmicutes;c__Clostr	0.011102703	k__Bacteria;p__Proteobacteria;c__Gam
k__Bacteria;p__Firmicutes;c__Bacilli	0.049643243	k__Bacteria;p__Firmicutes;c__Clostridi
k__Bacteria;p__Actinobacteriota;c__	0.031940541	k__Bacteria;p__Firmicutes;c__Clostridi
k__Bacteria;p__Proteobacteria;c__Ga	0.039859459	k__Bacteria;p__Bacteroidota;c__Bacter
k__Bacteria;p__Firmicutes;c__Clostr	0.011513514	k__Bacteria;p__Proteobacteria;c__Alph
k__Bacteria;p__Actinobacteriota;c__	0.023637838	k__Bacteria;p__Firmicutes;c__Clostridi
k__Bacteria;p__Firmicutes;c__Clostr	0.001843243	k__Bacteria;p__Firmicutes;c__Bacilli;o_
k__Bacteria;p__Firmicutes;c__Bacilli	0.031249407	k__Bacteria;p__Desulfobacterota;c__De
k__Bacteria;p__Verrucomicrobiota;c_	0.031249407	k__Bacteria;p__Actinobacteriota;c__Co
k__Bacteria;p__Myxococcota;c__Pol	0.044232432	k__Bacteria;p__Firmicutes;c__Negativi
k__Bacteria;p__Actinobacteriota;c__	0.041994595	k__Bacteria;p__Firmicutes;c__Clostridi
k__Bacteria;p__Firmicutes;c__Clostr	0.031249407	k__Bacteria;p__Firmicutes;c__Bacilli;o_
k__Bacteria;p__Firmicutes;c__Clostr	0.006524324	k__Bacteria;p__Firmicutes;c__Clostridi
k__Bacteria;p__Firmicutes;c__Bacilli	0.004275676	k__Bacteria;p__Firmicutes;c__Bacilli;o_
k__Bacteria;p__Firmicutes;c__Clostr	0.037043243	k__Bacteria;p__Firmicutes;c__Clostridi
k__Bacteria;p__Proteobacteria;c__Al	0.043227027	k__Bacteria;p__Firmicutes;c__Clostridi
k__Bacteria;p__Proteobacteria;c__Al	0.031249407	k__Bacteria;p__Firmicutes;c__Clostridi
k__Bacteria;p__Actinobacteriota;c__	0.040891892	k__Bacteria;p__Firmicutes;c__Bacilli;o_
		k__Bacteria;p__Firmicutes;c__Clostridi
		k__Bacteria;p__Firmicutes;c__Clostridi
		k__Bacteria;p__Proteobacteria;c__Gam
		k__Bacteria;p__Proteobacteria;c__Gam
		k__Bacteria;p__Firmicutes;c__Clostridi
		k__Bacteria;p__Proteobacteria;c__Gam
		k__Bacteria;p__Actinobacteriota;c__Ac
		k__Bacteria;p__Proteobacteria;c__Alph
		k__Bacteria;p__Bacteroidota;c__Bacter
		k__Bacteria;p__Firmicutes;c__Clostridi
		k__Bacteria;p__Firmicutes;c__Clostridi
		k__Bacteria;p__Firmicutes;c__Bacilli;o_
		k__Bacteria;p__Proteobacteria;c__Gam
		k__Bacteria;p__Proteobacteria;c__Gam
		k__Bacteria;p__Verrucomicrobiota;c__
		k__Bacteria;p__Myxococcota;c__Polya
		k__Bacteria;p__Actinobacteriota;c__Ac
		k__Bacteria;p__Proteobacteria;c__Gam
		k__Bacteria;p__Firmicutes;c__Clostridi
		k__Bacteria;p__Firmicutes;c__Bacilli;o_
		k__Bacteria;p__Firmicutes;c__Clostridi
		k__Bacteria;p__Firmicutes;c__Bacilli;o_
		k__Bacteria;p__Bacteroidota;c__Bacter
		k__Bacteria;p__Proteobacteria;c__Gam
		k__Bacteria;p__Firmicutes;c__Bacilli;o_
		k__Bacteria;p__Actinobacteriota;c__Co
		k__Bacteria;p__Proteobacteria;c__Alph

k__Bacteria;p__Proteobacteria;c__Alph[illegible]
k__Bacteria;p__Firmicutes;c__Clostridi[illegible]

p.value
0.035743455
0.013439791
0.002136126
0.04613089
0.023649215
0.031801047
0.007340314
0.006680628
0.046502618
0.010994764
0.00182199
0.043816754
0.048062827
0.006015707
0.027717277
0.004397906
0.000549738
0.026575916
0.030015707
0.049303665
0.001272251
0.036251309
0.046157068
0.032617801
0
0.000157068
0.021649215
0.009193717
0.006628272
0.005376963
0.040418848
0.011554974
0.028748691
0.022790576
0.036722513
0.032921466
0.001884817
0.001570681
0.023570681
0.013471204
0.018356021
0.031249407
0.04404712
0.041921466
0.012984293
0.017041885
0.012287958
0.001324607
0.031249407
0.04860733
0.014
0.038376963
0.003104712
0.04321466

0.031249407
0.031249407
